# Studying the Accuracy and Function of Different Thermometry Techniques for Measuring Body Temperature

**DOI:** 10.3390/biology10121327

**Published:** 2021-12-15

**Authors:** Aaron James Mah, Leili Ghazi Zadeh, Mahta Khoshnam Tehrani, Shahbaz Askari, Amir H. Gandjbakhche, Babak Shadgan

**Affiliations:** 1 Implantable Biosensing Laboratory, ICORD, Vancouver, BC V5Z 1M9, Canada; ghazi@icord.org (L.G.Z.); mkhoshna@alumni.uwo.ca (M.K.T.); shahbaz.askari@gmail.com (S.A.); babak.shadgan@ubc.ca (B.S.); 2 Department of Pathology & Laboratory Medicine, University of British Columbia, Vancouver, BC V6T 1Z7, Canada; 3 Department of Orthopedics, University of British Columbia, Vancouver, BC V6T 1Z7, Canada; 4 Department of Electrical Engineering, University of British Columbia, Vancouver, BC V6T 1Z7, Canada; 5 Section on Analytical and Functional Biophotonics, National Institute of Child Health and Human Development, Rockville, MD 20847, USA; gandjbaa@mail.nih.gov

**Keywords:** body temperature, remote monitoring, biosensor, thermometry, infrared thermometer, digital thermometer, tympanic thermometer, zero heat flux thermometer, infrared thermography

## Abstract

**Simple Summary:**

Core body temperature can provide a method for the early diagnosis of viral infections such as COVID-19. The current pandemic has highlighted the need for an accurate method for body temperature screening. The purpose of this study was to determine which thermometry technique is the most accurate for the regular measurement of body temperature. We compared seven different commercially available thermometers with a gold standard medical-grade thermometer. Our study showed that not all temperature monitoring systems are equal, and suggested that tympanic thermometers are the most accurate commercially available system for the regular measurement of body temperature. Tympanic thermometers can help individuals with regular self-assessment of their body temperature, which is a useful tool for lowering the spread of infectious diseases such as COVID-19.

**Abstract:**

The purpose of this study was to determine which thermometry technique is the most accurate for regular measurement of body temperature. We compared seven different commercially available thermometers with a gold standard medical-grade thermometer (Welch-Allyn): four digital infrared thermometers (Wellworks, Braun, Withings, MOBI), one digital sublingual thermometer (Braun), one zero heat flux thermometer (3M), and one infrared thermal imaging camera (FLIR One). Thirty young healthy adults participated in an experiment that altered core body temperature. After baseline measurements, participants placed their feet in a cold-water bath while consuming cold water for 30 min. Subsequently, feet were removed and covered with a blanket for 30 min. Throughout the session, temperature was recorded every 10 min with all devices. The Braun tympanic thermometer (left ear) had the best agreement with the gold standard (mean error: 0.044 °C). The FLIR One thermal imaging camera was the least accurate device (mean error: −0.522 °C)**.** A sign test demonstrated that all thermometry devices were significantly different than the gold standard except for the Braun tympanic thermometer (left ear). Our study showed that not all temperature monitoring techniques are equal, and suggested that tympanic thermometers are the most accurate commercially available system for the regular measurement of body temperature.

## 1. Introduction

Core body temperature is a vital indicator of health and illness, and is often considered a determining factor in the diagnosis and treatment of infection. It is defined as the blood temperature that bathes the thermoregulatory receptor in the hypothalamus [1]. Healthy individuals typically have a core body temperature between 36.5 °C to 37.5 °C, a temperature range that is essential for the metabolic processes of the body. An increase from these values may indicate a fever, which is a common symptom of infection [2]. Therefore, it is extremely important for measurement devices to be accurate and precise when determining body temperature. Invasive and complex techniques such as pulmonary artery catheters offer direct measurement of core body temperature and are regarded as the gold standard for determining body temperature. Other semi-invasive methods such as esophageal or rectal thermometry may provide close to core temperature measurements. However, the invasive and inconvenient nature of these methods makes them difficult to use outside of hospital settings [3].

Peripheral thermometers estimate core body temperature non-invasively at measurement sites such as the mouth, ear canal, and forehead. These devices offer an advantage over invasive thermometers due to their fast measurement speed and convenient access to measurement location [4]. However, estimating core temperature through the skin surface can be difficult, as skin temperature is lower than core body temperature and can be influenced by external factors such as ambient temperature, peripheral blood perfusion and remoteness of the measurement site [3,5]. The oral temperature may better indicate the core body temperature; however, its readings can be influenced by eating, drinking and smoking, and accurate measurement takes longer [6]. Glass mercury thermometers have historically been accepted as the gold standard for measuring body temperature non-invasively. However, the dangers of breakage and potential toxicity from mercury exposure have led to the decline of glass mercury thermometers in developed countries [7]. As a result, the use of infrared and digital thermometers has grown significantly over the last decade [3].

Commercially available devices use different techniques; however, all offer non-invasive estimates of core body temperature. These include infrared forehead thermometers, infrared tympanic thermometers, temporal artery thermometers, digital sublingual thermometers, zero heat flux thermometers, and thermal imaging cameras. Despite the abundance of temperature measuring devices available, there is uncertainty regarding the accuracy of non-invasive thermometers to detect fever [3]. Additionally, data that compare different peripheral thermometers for fever screening are limited. Understanding the most accurate non-invasive temperature measurement device can be exceptionally beneficial in high-risk individuals and during epidemic and pandemic viral outbreaks such as COVID-19.

### Objectives

The primary objective of this study is to determine the accuracy of seven commercially available thermometer systems compared with a current gold standard medical-grade thermometer system. The devices that we will compare include four digital infrared thermometers, one digital sublingual thermometer, one zero heat flux thermometer, and one infrared thermal imaging camera. We will also study and compare other features of these thermometers, including reproducibility, measurement speed, device cost, and data recordability. The outcomes of this study will help individuals choose the most accurate thermometry device for the self-assessment of body temperature.

## 2. Methods

In this study, we examined the accuracy and reliability of several commonly used thermometry techniques to detect changes of body core temperature in response to a body temperature manipulation protocol.

### 2.1. Selected Thermometer Systems

**Figure 1:**
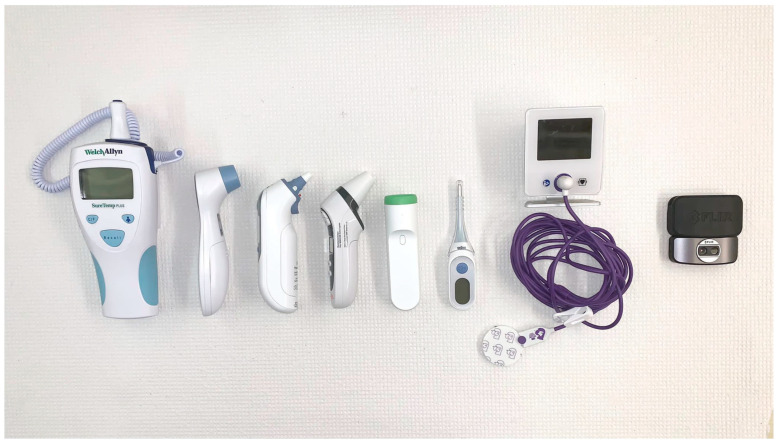
Selected thermometry devices. Left to right: Welch-Allyn digital sublingual, Wellworks infrared forehead, Braun infrared tympanic, MOBI infrared forehead, Withings temporal artery, Braun digital sublingual, 3M zero heat flux, FLIR One thermal imaging camera.

Seven commercially available thermometer systems, depicted in Figure 1, were compared with a clinical-grade, FDA and Health Canada-approved [8] digital thermometer (Welch-Allyn SureTemp Plus Electronic Thermometer Model 692, Welch Allyn Inc., Skaneateles Falls, NY, USA) as the gold standard system. The gold standard thermometer is commonly used in the emergency room in hospitals in Canada. Table 1 summarizes the main characteristics of each thermometer system. It should be noted that in choosing these thermometers, the focus was on the technique that they use for measuring temperature, and specific brands were not targeted. Furthermore, the devices used in this study are non-invasive, commercially available thermometers that can be used for regular temperature measurement.

#### 2.1.1. Infrared Forehead Thermometer

Infrared forehead thermometers take temperature measurements a short distance from the frontal bone without contacting the skin. These devices convert the infrared radiation emitted from the forehead to an electrical signal, which is then used to determine a temperature reading [2]. Many infrared thermometers use algorithms to convert the peripheral temperature measurement to the core temperature of the subject. However, these algorithms are not consistent between products, leading to variability between devices [4]. The forehead thermometer offers an advantage over other thermometers due to the non-contact nature of the device, which allows measurements to be taken quickly with no cleaning between individuals. This has made the infrared forehead thermometer increasingly popular for self-assessment and mass screening during situations such as the COVID-19 pandemic [2].

#### 2.1.2. Infrared Tympanic Thermometer

Infrared tympanic thermometers (ITTs) detect infrared radiation emitted from the tympanic membrane and convert it into an electric signal, which can then be interpreted as a temperature reading [2]. Tympanic thermometers are popular due to their fast measurement speed and easy access to the measurement location, making them a viable option as a temperature screening device. The contact nature of tympanic thermometers requires either cleaning of the device between subjects or using disposable sheaths to cover the thermometer probe. Therefore, it is not a practical method for mass screening.

#### 2.1.3. Infrared Temporal Artery Thermometer

Temporal artery thermometers (TATs) record temperature by slowly moving the device from the centre of the forehead to the lateral hairline to detect infrared radiation emitted from the skin over the superficial temporal artery [9]. The thermometer takes up to 1000 readings per second during the course of the measurement and reports the highest temperature [2]. The device then uses an algorithm to adjust for ambient temperature and calculate core temperature [10]. TATs provide an advantage over contact thermometers as they do not require disposable covers, thus offering a cheaper and faster alternative for temperature screening [9].

#### 2.1.4. Digital Sublingual Thermometer

Digital sublingual thermometers, also known as oral thermometers, measure temperature under the tongue in the sublingual pocket and are popular in clinical settings [11]. Many digital thermometers contain thermistors, which change resistance with the temperature. This allows digital thermometers to measure small changes in temperature over a short range [4]. Oral thermometers require a disposable cover or method for disinfection between uses due to the risk of cross-infection between subjects [6].

#### 2.1.5. Zero Heat Flux Thermometer

Zero heat flux (ZHF) thermometry is a novel alternative technique to peripheral thermometry that provides a non-invasive estimate of core body temperature. ZHF thermometers contain a sensor that is composed of a thermal insulator covered by an electric heater [12]. The sensor is slowly heated until its temperature is equal to the skin temperature, creating an isothermal pathway from the core body to the skin surface. Once the sensor is sufficiently heated to reach thermal equilibrium, a zero heat flux condition is established, and the device is able to measure temperature approximately 1–2 cm below the skin surface [12]. This system is also designed for continuous monitoring of core body temperature in clinical settings.

#### 2.1.6. Infrared Thermal Imaging Camera

Infrared thermal cameras are non-invasive body temperature measurement devices in which the camera and operator can be located some distance away from the subject. Thermal cameras function by detecting the infrared radiation emitted from human skin and converting it into an electrical signal, which is used to display a body temperature profile [13]. Infrared thermography displays body temperature distribution over a large surface, including any hot or cold patterns [14]. This makes infrared thermography feasible as a method for detecting individuals with an elevated temperature and established fever. This method is commonly used for mass screening in places such as airports, seaports, border crossings, and other places such as shopping centers and hospital entrances [14].

### 2.2. Participants

A cohort of 30 young healthy adults (28.3 ± 9.4 years old, 14 male) from the Vancouver Lower Mainland was recruited for participation in the study. Participants were included in the study if they were healthy adults between the ages of 17–65 and excluded if they had an acute medical condition, were taking antipyretic medication, had allergic reactions to cold water, or were pregnant. The experiment was conducted at ICORD in Blusson Spinal Cord Centre in Vancouver. The ambient temperature of the study location was 23 °C and the relative humidity was 55.0%. The Clinical Research Ethics Board at the University of British Columbia approved the protocol of this study, and all participants provided informed consent at the beginning of the data collection session.

### 2.3. Experimental Protocol

**Figure 2:**
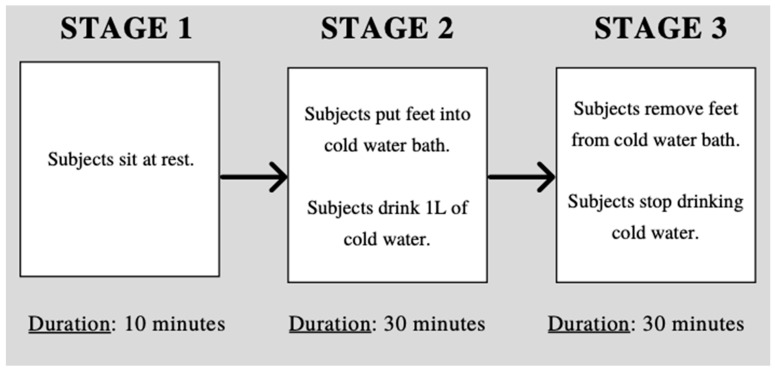
Flowchart describing the experimental protocol.

The protocol was designed to evaluate the accuracy of each thermometer while changing the body temperature of the subject. Figure 2 shows the study protocol flowchart. Each measurement was taken by two trained research assistants. Before beginning the experiment, participants were instructed to sit in a resting position for 15 min to ensure that external factors such as outside temperature and increased heart rate did not influence the thermometer systems. Measurements were taken twice in short succession with each device and the average of the two measurements was recorded. The order of temperature measurements remained the same for every participant and began with the gold standard (Welch-Allyn, Auburn, NY, USA), followed by the Braun digital sublingual, Withings temporal artery, Wellworks infrared forehead, MOBI infrared forehead, Braun infrared tympanic, 3M zero heat flux and FLIR One thermal imaging camera. The gold standard system measured temperature under the tongue in the sublingual pocket. Measurements using the tympanic thermometer (Braun Thermoscan 7 [Braun, Frankfurt, Germany]) were taken in both ears. The infrared imaging measurements using the FLIR One Thermal Imaging Camera were taken from both close range (10 cm) and long range (50 cm). For devices that required a disposable cover (Braun Thermoscan, Welch-Allyn SureTemp Plus [Welch-Allyn, Auburn, NY, USA]), a new cover was used for each measurement. The digital sublingual thermometer (Braun, Frankfurt, Germany) was sanitized using alcohol before use for each participant.

The first measurement was taken using each device with the participant sitting quiescent on a chair in a resting position to establish a baseline. After the baseline measurements had been taken, participants were instructed to submerge their feet in a 16 L cold-water bath for 30 min to lower their body temperature. Previous research has demonstrated the effect of cold-water immersion on lowering body temperature [15,16]. While remaining in the cold-water bath, participants were instructed to drink one liter of cold water (6.0 °C) gradually for 30 min to further decrease their body temperature. Participants were instructed to stop drinking cold water two minutes before the measurements were taken so that their oral temperature was not influenced by the presence of cold water. The initial temperature of the cold-water bath was approximately 20.1 °C. One scoop of ice (60 mL) was added at 5 min, 15 min and 25 min. After 5 min, the cold-water bath had an approximate temperature of 16.3 °C. After 15 min, the cold-water bath had an approximate temperature of 11.87 °C. After 25 min, the cold-water bath had an approximate temperature of 10.63 °C. A digital thermometer (FisherScientific Traceable Exursion-Trac Datalogging, [Waltham, MA, USA]) was used to monitor the temperature of the cold-water bath. The temperature of the cold-water bath was adjusted based on the amount of ice added to the water (60 mL) at 5, 15 and 25 min. The original target temperatures were determined experimentally based on how much ice could be added to the cold-water bath while being tolerable for 30 min for the subjects. The temperature of the cold-water bath was recorded after the ice was added to the water, so there was no rationale behind the target temperatures; however, we were consistently within ±0.3 °C of these target temperatures for each subject. Temperature data were recorded at a 10-min interval while participants were submerged in the cold-water bath. After 30 min, participants were asked to remove their feet from the cold-water bath and stop drinking cold water. Body temperature continued to be collected while participants were at rest for an additional 30 min at a 10-min interval while body temperature returned to baseline. During the 70-min experiment, body temperature was recorded at seven different time points with each device.

### 2.4. Statistical Methods

A chi-square goodness of fit test was conducted on all measurements at different time points to determine if the data were modelled by a normal distribution. The results indicated that the measurements of every device were not normally distributed. Therefore, a sign test was conducted to determine whether there were significant differences between each of the thermometer systems and the gold standard. A 95% confidence interval was calculated at each time point to determine which devices recorded a mean temperature within 0.1 °C of the gold standard. Bland–Altman plots were created to demonstrate the agreement between each device and the gold standard across the temperature range. Data analysis, including the calculation of standard deviation, mean difference and statistical tests, were carried out using Microsoft Excel 2021 and MATLAB 2021.

## 3. Results

### 3.1. Mean Temperature Difference

**Figure 3:**
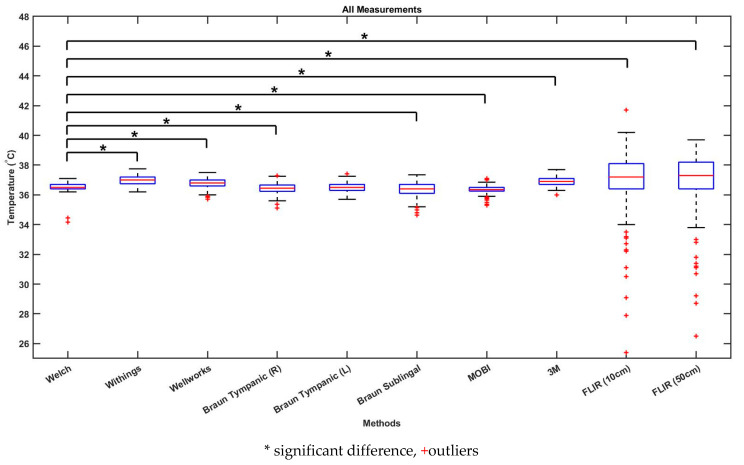
Boxplot of all temperature readings.

**Figure 4:**
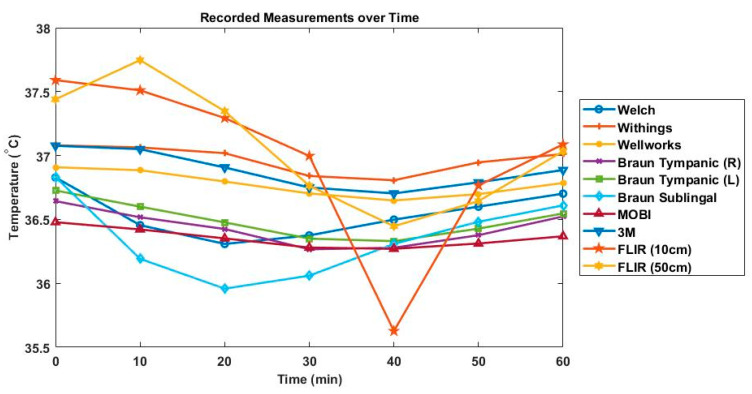
Body temperature changes over time.

Table 2 shows the mean differences, standard deviation and 95% confidence intervals between each device and the gold standard. The Braun tympanic thermometer (left ear) had the lowest mean difference, at −0.044 °C, and the FLIR One thermal imaging camera (50 cm) had the highest mean difference, +0.522. Figure 3 shows a boxplot of all temperature readings for each device. Figure 4 displays the changes in body temperature using all devices during the 70-min experiment.

### 3.2. Statistical Analysis

**Figure 5:**
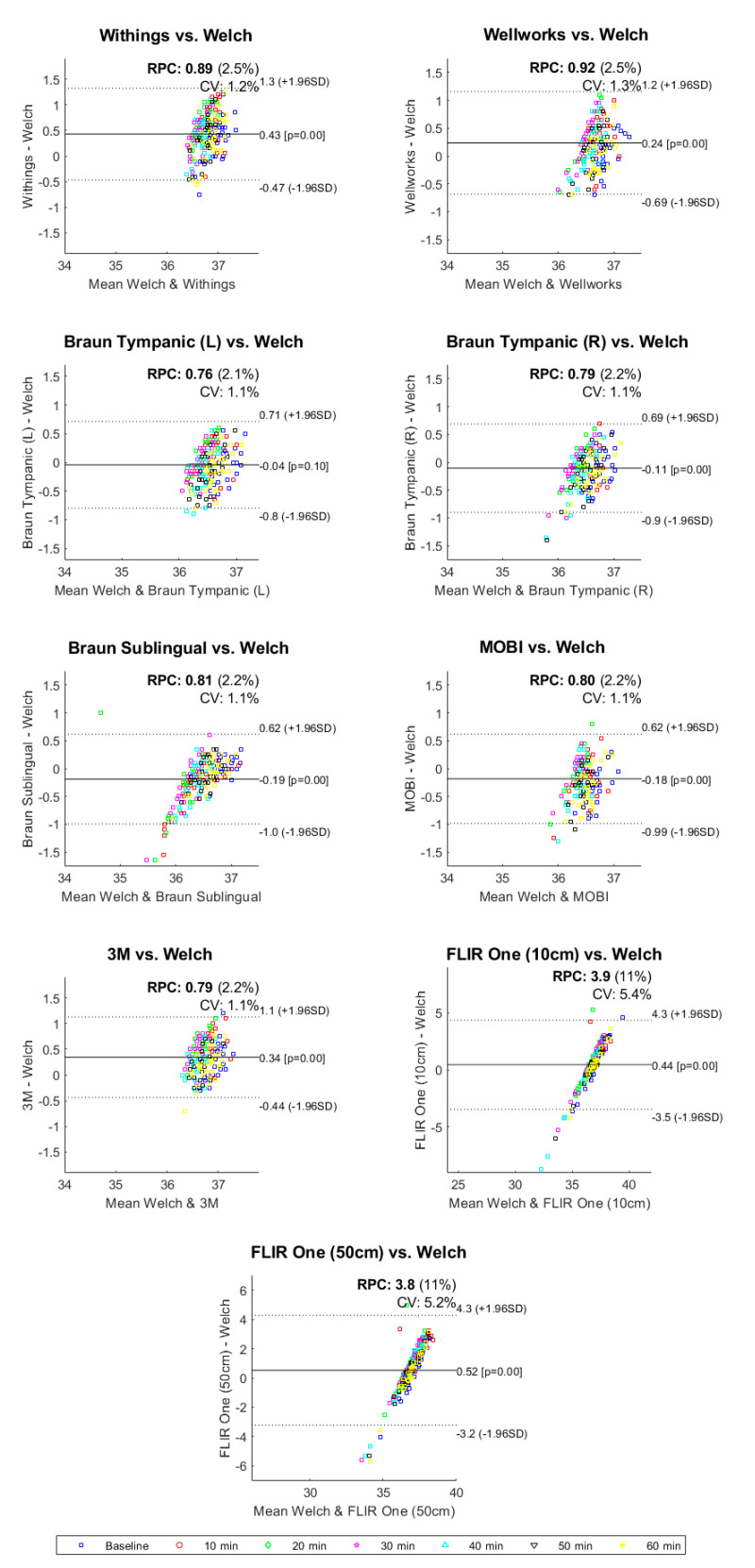
Bland−Altman plots for all thermometers vs. the gold standard.

Table 3 shows the results of the sign test between each device and the gold standard. Every device demonstrated a significant difference (*p* < 0.05) in temperature from the gold standard except for the Braun tympanic thermometer (left ear). Figure 5 displays the Bland–Altman plots of all thermometers compared to the gold standard. The difference between the gold standard and each device (y-axis) is plotted as a function of the mean of the two measurements (x-axis). The Bland–Altman plot for the Braun tympanic thermometer (left ear) shows that 95% of the measurements presented differences between −0.8 °C and +0.71 °C, with a mean difference of −0.04 °C. These were the smallest limits of agreements for all devices, indicating higher accuracy compared to the gold standard. The Braun tympanic thermometer (right ear) also demonstrated a high level of accuracy, with a mean difference of −0.11 °C, although with greater variability at lower temperatures. The Bland–Altman plot indicates that the Withings temporal artery, Wellworks infrared forehead and 3M zero heat flux thermometer overestimated body temperature, with upper limits of agreement greater than 1.0 °C. The Braun sublingual and MOBI infrared forehead thermometer underestimated body temperature. The FLIR One thermal imaging camera had the largest limits of agreement.

## 4. Discussion

Early detection of fever, particularly in high-risk individuals such as neonates, the elderly and people with immune deficiency and chronic conditions, can improve the effectiveness of treatment options. Furthermore, early detection of fever by vigilant screening and regular body temperature measurement is critical during epidemic and pandemic viral outbreaks. Following the SARS epidemic in 2003, several countries adopted body temperature measurement as a rapid and non-contact method for fever screening at border crossings. The current COVID-19 pandemic further highlighted the need for individuals to have access to an accurate temperature screening system to prevent the spread of infection. Like many infectious diseases, the early detection and treatment of COVID-19 are paramount to decreasing the transmission of the disease and increasing the chances of easier recovery. While early treatment of COVID-19 has been linked to improved patient outcomes and a decrease in hospitalizations, long-term benefits of early treatment include reducing chronic effects of the disease and a shortened period of infectiousness, leading to lower rates of transmission [17]. During the current pandemic, thermal screening via infrared thermal imaging systems and non-contact infrared thermometers (NCITs) are common in public areas such as schools, airports, grocery stores and hospitals [18]. These devices provide an advantage over conventional temperature measurement devices due to their fast measurement times and non-contact nature, reducing the risk of cross-infection between subjects [19]. However, it is unclear if NCITs provide an accurate and precise measurement of body temperature in adults [2,14]. When considering an ideal measurement device for body temperature screening, accuracy must be heavily considered.

In this study, the Braun infrared tympanic thermometer (left ear) demonstrated the highest accuracy when compared with the gold standard. The mean difference of −0.044 °C with the gold standard meets the criterion that an ideal thermometer should be accurate within +/−0.1 °C, as described by Moran and Mendel [20]. Our results are supported by previous studies that demonstrate the strong accuracy of various tympanic thermometers [21,22,23]. Measurements using the Braun tympanic thermometer were taken in both the left and right ear. There was a small temperature difference between the left and right ear (mean error: −0.06 °C), suggesting that temperature readings between ears are not equal. However, a temperature difference less than 0.2 °C is typically considered clinically insignificant [7]. A sign test resulted in a *p* value of 0.0364, demonstrating a significant difference between both ears. Previous studies have reported a temperature difference between left and right ears [24,25]. However, other studies have reported no significant difference between the left and right ears [22,23,26]. Furthermore, White et al. [22] suggested that differences in temperature between the left and right ears may be due to poor measurement technique, or to cerumen blocking the tympanic membrane. Structural variations and differences between the right and left ears may also explain this observation [11]. To reduce measurement error for tympanic thermometers, proper care must be taken to follow standard measurement technique. Before the measurement, gently pull back the pinna to sufficiently straighten the external auditory meatus. Then, insert the thermometer probe into the ear canal as far as it will comfortably go to create a seal. Only press the start button once the probe has been inserted, and do not remove it until the device beeps and a temperature is visible on the display [1,9]. The results of our study suggest that tympanic thermometry is suitable for the regular measurement of core body temperature. Despite using a different measurement technology than the gold standard, the infrared tympanic thermometer demonstrated the smallest mean difference of all thermometer devices. Furthermore, the Braun infrared tympanic thermometer used in this study is commercially available, with a current price of CAD 58.00. Due to the slight differences between the right and left ear observed in this study, we recommend taking measurements in the same ear when using a tympanic thermometer for self-assessment of body temperature.

The Braun sublingual thermometer demonstrated good accuracy compared to the gold standard during the baseline (mean error: +0.003 °C) and recovery stage (mean error: −0.092 °C). However, during the body temperature changes, the mean temperature dropped below the gold standard by as much as −0.315 °C. This may be due to the ingestion of cold water, which could impact sublingual temperature measurement. The influence of ingesting food or drink has been reported by previous studies and is an important factor when determining the accuracy of oral thermometers [6,11,20]. There are mixed reports describing the accuracy of sublingual thermometers. Our results are consistent with a previous study indicating that oral thermometry frequently underestimates core body temperature when compared with a gold standard rectal thermometer [27]. Additional studies have reported that conventional oral thermometers may fail to measure core body temperature [9,28,29,30]. The Braun sublingual thermometer was the least expensive device used in this study, with a current price of CAD 24.00. Due to the significant difference between the digital sublingual thermometer and the gold standard, in addition to the influences of external factors, this thermometry technique is not recommended for sensitive and regular measurement of body temperature.

The Wellworks infrared forehead thermometer demonstrated good accuracy during the baseline (mean error: +0.079 °C) and recovery phase (mean error: +0.083 °C). However, during the body temperature changes the thermometer was not as responsive to lowering the core body temperature, with a mean error of +0.489 °C after 20 min in the cold-water bath. The MOBI infrared forehead thermometer was also tested during this study. This thermometer demonstrated more accurate readings than the Wellworks infrared forehead thermometer when compared to the gold standard (mean difference: −0.184 °C). However, this device underestimated temperature during the baseline and recovery readings. Furthermore, the temperature readings did not change significantly between the baseline and cold-water phase (−0.1983 °C), indicating that this device may not be as sensitive to changes in body temperature. The results of this study are consistent with previous research indicating that infrared forehead thermometers do not provide an accurate representation of core body temperature [14,31,32]. An additional study reported that infrared forehead thermometers are insufficient for detecting fever, which is a crucial component in body temperature screening [33]. Furthermore, previous research has reported that infrared forehead thermometers can be influenced by a wide range of factors including perspiration, ambient temperature and humidity, body activity, and exercise [4]. The Wellworks infrared forehead thermometer has a price of CAD 50.00 and the MOBI infrared forehead thermometer has a price of CAD 29.00 at the time of this publication. Due to the significant temperature differences with the gold standard thermometer, forehead infrared thermometers are not recommended for regular measurement of body temperature.

**Figure 6:**
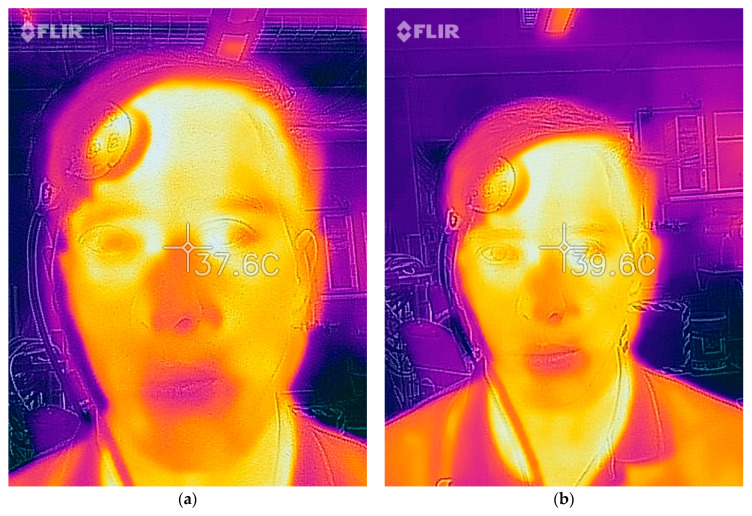
FLIR One Thermal Imaging Camera from 10 cm (**a**) and 50 cm (**b**).

The FLIR One thermal imaging camera was operated from both close range (10 cm) and long range (50 cm), demonstrated in Figure 6. This thermal imaging camera proved to be the least accurate device and there was a considerable amount of variability between measurements, as demonstrated by the large range of temperatures seen in Figure 3 and Figure 4. This study supports previous research suggesting that thermal imaging cameras are inaccurate for body temperature screening [14]. The FLIR One thermal imaging camera has a current price of CAD 530.00. The results of the current study suggest that thermal imaging cameras are not accurate for the regular screening of body temperature. However, infrared thermal imaging cameras are relatively new for the use of body temperature screening. As a result, there is limited research on the accuracy of these devices. More research needs to be done to determine if advanced thermal imaging cameras are suitable for body temperature screening.

The Withings temporal artery thermometer was consistently higher than the gold standard at every time point. Our results are supported by previous studies indicating the poor accuracy of temporal artery thermometers [9,10,30,34]. Previous research has reported that variance in conditions such as temperature and humidity in addition to external factors such as medication and physical activity can cause inaccurate temperature readings [14]. Normal anatomical variations of the temporal artery’s location and size in different individuals might be a causative factor of this technique’s lower accuracy. The Withings temporal artery thermometer has a current price of CAD 130.00. Due to the temporal artery thermometer consistently overestimating core body temperature, this device is not recommended for regular temperature screening.

Throughout the 60-min experiment, the 3M zero heat flux (ZHF) thermometer was slow to respond to changes in body temperature. While the baseline reading was relatively close to the gold standard (mean error: +0.248 °C), the ZHF thermometer did not adjust to the reduction in body temperature during the cold-water phase. This is reflected in a mean error of +0.595 °C after 10 min in the ice bath and +0.598 °C after 20 min. The results of this study agree with previous research that question the accuracy of ZHF thermometers for snapshot measurement of core body temperature [35]. Furthermore, the 3M zero heat flux thermometer is the most expensive device used in this study, with a current price of CAD 1100.00. A primary issue with ZHF thermometers is the amount of time that it takes for the device to reach equilibrium and show an accurate temperature reading. During the baseline measurement, the device took up to 10 min after the placement of the sensor to display the core body temperature. Several studies have, however, indicated that ZHF thermometers are suitable for continuous monitoring of patient’s temperature at the bedside [5,36,37]. While continuous temperature monitoring may be useful for a clinical setting, the long wait time needed to display a temperature reading makes this system unsuitable for use as a rapid temperature screening device.

While every device tested in this study is available for use by the general population, improper placement of the thermometer probes may contribute to measurement error. Digital sublingual thermometers require the thermometer probe to be positioned under the tongue, and provide the highest accuracy when placed in the left or right sublingual pocket. Differences in probe placement may contribute to temperature variation [20]. Infrared tympanic thermometers obtain the highest temperature from the tympanic membrane, and temperature decreases with distance away from the membrane [11]. This may contribute to variability between measurements when using infrared tympanic thermometers. Non-contact devices such as infrared forehead thermometers and temporal artery thermometers have an optimal distance of 2–5 cm from the skin. To obtain an accurate temperature reading, the thermometer probe should be held a consistent distance from the skin surface for each measurement. Additionally, hair should not be covering the forehead [9]. Thermal imaging cameras typically contain the whole face within the frame when recording temperature. However, it is important to obtain consistent temperature readings from a specific region of the face. The inner-eye region is common for thermal imaging cameras [13].

We recognize limitations in our study, including our sample size being limited to 30 participants. Furthermore, only healthy individuals were included in this study. More research needs to be done to ensure that different patient cohorts are represented. A similar study must also be conducted in children and neonates. The study protocol included measurements taken over a 70-min period. This short time frame may not account for larger body temperature changes that could be seen in a longer study protocol.

An additional limitation of our study is the use of an oral thermometer as a gold standard. To obtain a direct measurement of core body temperature, a pulmonary artery catheter is required in order to ensure that no external factors influence the temperature reading. Additional invasive techniques such as rectal or esophageal thermometers can also provide a close estimation of core body temperature. However, the volunteer participants in this study were all healthy adults and as a result, the use of invasive thermometers was not preferred. Therefore, we used a surrogate method which is clinically accurate within ±0.1 °C and is currently used in the emergency wards of hospitals in Canada. Furthermore, the experimental protocol was designed to limit the influence of the external environment.

## 5. Conclusions

We demonstrated that tympanic thermometers are the most accurate commercially available system for the regular measurement of core body temperature. Furthermore, the results of this study suggest that the tympanic thermometer provided a more accurate body temperature reading when used in the left ear. The temporal artery thermometer, infrared forehead thermometer, digital sublingual thermometer, zero heat flux thermometer, and thermal imaging camera all demonstrated a significant temperature difference from the gold standard. Tympanic thermometers can help individuals with regular self-assessment of their body temperature, which is a useful tool in lowering the spread of infectious diseases such as COVID-19.

## Figures and Tables

**Table 1:** Characteristics of selected thermometry devices.

Device	Company	Model Number	Meas. Site	Meas. Distance	Meas. Speed	Data Recordability	Clinical Accuracy	Price (CAD)
SureTemp Plus *	Welch-Allyn	692	Oral	Contact	6 s	No	±0.1 °C	$501
Temporal Artery	Withings	SCT01	Forehead	2–5 cm	3 s	Yes	±0.2 °C	$130
Infrared Forehead	Wellworks	FDIR-V16	Forehead	2–5 cm	1 s	No	±0.3 °C	$50
Infrared Tympanic	Braun	IRT6520	Ear	Contact	1 s	No	±0.2 °C	$58
Digital Sublingual	Braun	PRT2000	Oral	Contact	8 s	No	±0.1 °C	$24
Infrared Forehead	MOBI	70121	Forehead	2–5 cm	1 s	No	±0.2 °C	$29
Zero Heat Flux	3M	3700	Forehead	Contact	Continuous (After equilibrium)	No	±0.23 °C	$1100
Thermal Imaging Camera	FLIR One	FLIR One Pro	Face	10 cm/50 cm	1 s	Yes	±3 °C	$530

* Gold Standard.

**Table 2:** Mean difference, standard deviation and 95% confidence interval between each device and the gold standard.

	Withings	Wellworks	Braun Tympanic (R)	Braun Tympanic (L)	Braun Sublingual	MOBI	3M	FLIR One (10 cm)	FLIR One (50 cm)
Mean Difference	+0.429	+0.237	−0.106	−0.044	−0.189	−0.184	+0.342	+0.443	+0.522
SD	0.359	0.315	0.301	0.279	0.331	0.283	0.311	1.333	1.334
CI_95_	±0.129	±0.113	±0.108	±0.099	±0.119	±0.101	±0.111	±0.477	±0.478

Note: CI_95_ = 95% confidence interval.

**Table 3:** Results of the sign test between each device and the gold standard.

	Withings	Wellworks	Braun Tympanic (R)	Braun Tympanic (L)	Braun Sublingual	MOBI	3M	FLIR One (10 cm)	FLIR One (50 cm)
*p* value	0.0000	0.0000	0.000	0.1747 *	0.0000	0.0000	0.0000	0.0000	0.0000

* no significant difference, Note: significance level: *p* < 0.05.

## Data Availability

The data presented in this study are available on request from the corresponding author. The data are not publicly available due to privacy and ethical concerns.
